# Identification and evaluation of new reference genes in *Gossypium hirsutum *for accurate normalization of real-time quantitative RT-PCR data

**DOI:** 10.1186/1471-2229-10-49

**Published:** 2010-03-21

**Authors:** Sinara Artico, Sarah M Nardeli, Osmundo Brilhante, Maria Fátima Grossi-de-Sa, Marcio Alves-Ferreira

**Affiliations:** 1 Department of Genetics, Federal University of Rio de Janeiro-UFRJ Av Prof Rodolpho Paulo Rocco, s/n - Prédio do CCS Instituto de Biologia, 2oandar - sala A2-93, 219410-970 - Rio de Janeiro, RJ - Brasil; 2 Embrapa Genetic Resources and Biotechnology, Parque Estação Biológica - PqEB - Av W5 Norte (final) Caixa Postal 02372 - Brasília, DF - Brasil - 70770-900

## Abstract

**Background:**

Normalizing through reference genes, or housekeeping genes, can make more accurate and reliable results from reverse transcription real-time quantitative polymerase chain reaction (qPCR). Recent studies have shown that no single housekeeping gene is universal for all experiments. Thus, suitable reference genes should be the first step of any qPCR analysis. Only a few studies on the identification of housekeeping gene have been carried on plants. Therefore qPCR studies on important crops such as cotton has been hampered by the lack of suitable reference genes.

**Results:**

By the use of two distinct algorithms, implemented by *geNorm *and *NormFinder*, we have assessed the gene expression of nine candidate reference genes in cotton: *GhACT4, GhEF1α5, GhFBX6, GhPP2A1, GhMZA, GhPTB, GhGAPC2, GhβTUB3 *and *GhUBQ14*. The candidate reference genes were evaluated in 23 experimental samples consisting of six distinct plant organs, eight stages of flower development, four stages of fruit development and in flower verticils. The expression of *GhPP2A1 *and *GhUBQ14 *genes were the most stable across all samples and also when distinct plants organs are examined. *GhACT4 *and *GhUBQ14 *present more stable expression during flower development, *GhACT4 *and *GhFBX6 *in the floral verticils and *GhMZA *and *GhPTB *during fruit development. Our analysis provided the most suitable combination of reference genes for each experimental set tested as internal control for reliable qPCR data normalization. In addition, to illustrate the use of cotton reference genes we checked the expression of two cotton MADS-box genes in distinct plant and floral organs and also during flower development.

**Conclusion:**

We have tested the expression stabilities of nine candidate genes in a set of 23 tissue samples from cotton plants divided into five different experimental sets. As a result of this evaluation, we recommend the use of *GhUBQ14 *and *GhPP2A1 *housekeeping genes as superior references for normalization of gene expression measures in different cotton plant organs; *GhACT4 *and *GhUBQ14 *for flower development, *GhACT4 *and *GhFBX6 *for the floral organs and *GhMZA *and *GhPTB *for fruit development. We also provide the primer sequences whose performance in qPCR experiments is demonstrated. These genes will enable more accurate and reliable normalization of qPCR results for gene expression studies in this important crop, the major source of natural fiber and also an important source of edible oil. The use of bona fide reference genes allowed a detailed and accurate characterization of the temporal and spatial expression pattern of two MADS-box genes in cotton.

## Background

Gene expression analysis is increasingly important in many fields of biological research. Understanding patterns of expressed genes is crucial to provide insights into complex regulatory networks and will lead to the identification of genes relevant to new biological processes [1].

Reverse transcription real-time quantitative polymerase chain reaction (qPCR) is a robust method to study gene expression changes [2]. The main advantages of qPCR when compared to other experimental techniques used to evaluate gene expression levels, such as Northern blot hybridization and reverse transcription-polymerase chain reaction (RT-PCR), are its higher sensitivity, specificity, and broad quantification range of up to seven orders of magnitude [3]. Therefore, qPCR analysis has become the most common method for validating the whole-genome microarray data or a smaller set of genes and molecular diagnostics [4]. Although being extremely powerful technique, qPCR suffers from certain pitfalls, noteworthy the use of unreliable reference genes for the normalization step [5]. Normalization is necessary for the correction of non-specific variations, such as inaccurate quantification of RNA and problems in the quality of RNA that can trigger variable reverse transcription and PCR reactions. A number of strategies have been proposed to normalize qPCR data but normalization remains one of the most important challenges concerning this technique [5].

The expression of reference genes used for normalization in qPCR analysis should remain constant between the cells of different tissues and under different experimental conditions; otherwise, it can lead to erroneous results. Recent reports have demonstrated that some of the most well-known and frequently used reference genes are inappropriate for normalization in qPCR analysis due to expression variability [6-8]. The importance of reference genes for plant qPCR analysis has been recently emphasized even though the identification of these genes is quite laborious [9,10]. Microarray datasets can also be a rich source of information for selecting qPCR reference genes [6], but unfortunately, this tool is still not available for most of plant species, including cotton.

The classical housekeeping genes involved in basic cellular processes such as 18 S rRNA, ubiquitin, actin, β-tubulin, and glyceraldehyde-3-phosphate dehydrogenase have been recurrently used as internal controls for gene expression analysis in plant as they are supposed to have a uniform expression all samples and experimental conditions tested. However, several reports demonstrated that the transcript levels of these genes also vary considerably under different experimental conditions and are consequently unsuitable for gene expression studies [6,11]. Statistical algorithms such as *geNorm *[1], *NormFinder *[12] and *BestKeepe*r [13] have been developed for the evaluation of best suited reference gene(s) for normalization of qPCR data in a given set of biological samples. Recognizing the importance of reference genes in normalization of RT-qPCR data, various housekeeping genes have been evaluated for stable expression under specific conditions in various organisms. Many works have been carried on animal and human health [3,14] field that describe the identification of multiple reference genes for normalisation of qPCR data, but similar reports are scarce in plant research [4,15,16]. Czechowski *et al*. (2005) employed a new strategy for the identification of reference genes in *Arabidopsis thaliana*. Based on the microarray data of Affymetrix ATH1, several new reference genes were revealed in *Arabidopsis *[6]. Some of these genes have no previous information about function in *Arabidopsis *or any other organism. The list of new *Arabidopsis *reference genes revealed by Czechowski and collaborators was successfully employed to search reference genes in unrelated species such as *Vitis vinifera *by sequence homology [9]. Recently, our group was also successful in providing new reference genes for qPCR in *Coffea arabica *and *Brachiaria brizantia *using the same strategy employed in *V. vinifera *[17,18].

Cotton (*Gossypium *spp.) is the world's most important source of natural fiber and also an important source of edible oil [19]. Because of its unique reproductive developmental aspects and speciation history, *G. hirsutum *has attracted considerable scientific interest, not only among plant breeders and agricultural scientists, but also among taxonomists, developmental geneticists, and evolutionary biologists [20-24]. In spite of this, qPCR analyses in cotton are still hampered by the use of inappropriate references genes.

In this study, we report the validation of housekeeping genes to identify the most suitable internal control gene(s) for normalization of qPCR data obtained in different plant organs and floral verticils and also during flower and fruit development. In addition, to illustrate the usefulness of the new reference genes, we provided a detailed expression analysis of two MADS-box transcription factors in cotton, putative homologues of *Arabidopsis AGAMOUS *and *SEPALLATA3 genes*.

## Methods

### Plant Material

Experiments were performed using three-month old *Gossypim hirsutum *plants variety "BRS Cedro". Plants were grown under controlled temperature (21 ± 4°C) and natural photoperiod in Embrapa CENARGEM in Brasília (DF, Brazil). The organs used from cotton plants were flower buds, fruits, leaves, stems, branches, roots and floral meristem. We also included seven stages of flower development (flower buds with the following diameter sizes: 2, 4, 6, 7, 8, 10 and 12 mm) and four stages of fruit development (fruits with the following diameter sizes:10 to 15, 16 to 20, 21 to 30 and larger than 30 mm)[25]. The stages of flower and fruit and the respective major events of development are summarized in Additional file 1. In addition, floral organs (sepal, petal, stamen, carpel and pedicel) from 6 mm flower buds were dissected and harvested. The material was harvested from, at least, five different cotton plants to obtain one pool. The procedure was repeated with five distinct plants in order to obtain a second pool, the biological replicate. All samples were immediately frozen in liquid nitrogen and stored at -80°C until needed for RNA extraction.

### Total RNA isolation and cDNA synthesis

Frozen samples were ground to a fine powder in liquid nitrogen with a pestle and mortar. The total RNA extractions were performed from 100 mg of each macerate plant tissue in liquid nitrogen, using Invisorb Spin Plant RNA Mini kit (Invitek) according to the protocol of the manufacturer. Two other methods of RNA extraction were evaluated (Qiagen Plant RNA easy kit and Trizol), but the yields and DNA purity in our hands were unsatisfactory (data not shown). RNA concentration and purity were determined using a NanoDropTM Spectrophotometer ND-1000 (Thermo Scientific), and the integrity of RNA was also assessed by 1% agarose gel electrophoresis and ethidium bromide staining. The presence of contaminant DNA in the RNA samples was verified by PCR using primers spanning two exon and gel electrophoresis analysis. No fragments of genomic DNA were identified in all samples tested in this work (data not shown). The presence of spurious product of amplification caused by genomic DNA was also continuously checked by the verification of RT-qPCR dissociation profile. Both tests showed that the Invisorb Spin Plant RNA Mini kit efficiently removed contaminant DNA from the RNA samples. cDNAs were synthesized by adding 50 μM of Oligo(dT24V) primer and 10 mM of each deoxyribonucleoside 5'-triphosphate (dNTPs) to 1 μg of total RNA. This mixture was incubated at 65°C for five minutes, and briefly chilled on ice. First Strand Buffer, 20 mM of dithiothreitol (DTT) and 200 units of Superscript III (Invitrogen) were added to the prior mixture and the total volume (20 μL) was incubated at 50°C for 1 h following manufacturer's instructions. Inactivation of the reverse transcriptase was done by incubating the mixture at 70°C for 15 min and the cDNA solution was stored at -20°C.

### Real-time quantitative polymerase chain reaction (qPCR)

Eight of the nine putative cotton reference genes evaluated in this work, *GhACT4 *(actin gene family), *GhEF1α5 *(elongation factor 1-alpha), *GhFBX6 *(F-box family protein), *GhPP2A1 *(catalytic subunit of protein phosphatase 2A), *GhMZA *(clathrin adaptor complexes medium subunit family protein), *GhPTB *(polypyrimidine tract-binding protein homolog), *GhGAPC2 *(glyceraldehyde-3-phosphate dehydrogenase C-2), *GhβTUB3 *(β-tubulin), were selected according to their similarity to reference genes identified in *Arabidopsis *(Table 1) [6]. The sequences of possible *G. hirsutum *homologues were identified through a BLASTN against the database of the Green plant GB TAIR (*The A. thaliana Information Resource*, http://www.arabidopsis.org/). Only sequences that showed similarity higher than 1e-75 (E-value) were considered as putative homologous to the *Arabidopsis *genes and were selected for primer design. We also selected the gene encoding the poly-ubiquitin, *GhUBQ14*, commonly used in cotton for experiments of Northern blots and RT-qPCRs [26,27] (Table 1). Primers were designed with *Primer 3 *software [28] using as criterion amplified products from 80 to 180 bp with a Tm of 60 ± 1°C (primer sequences are shown in Table 1). Both candidate reference and MADS-box genes were amplified from cDNA. Melting curve and gel electrophoresis analysis of the amplification products confirmed that the primers amplified only a single product with expected size (data not shown). Primer sets efficiencies were estimated for each experimental set by *Miner *software [29], and the values were used in all subsequent analysis (Table 2 and Additional file 2). *Miner *software pinpoints the starting and ending points of PCR exponential phase from raw fluorescence data, and estimates primer set amplification efficiencies through a nonlinear regression algorithm without the need of a standard curve.

**Table 1:** Reference genes and their primer sequences that were selected for evaluation of expression stability during flower development in cotton (*Gossypium hirsutum*) for qPCR analysis, as the sequence of two genes of interest MADS-box.

*Gene abbreviation*	*Acession*	*A. thaliana ortholog locus*	*A. thaliana annotation*	*Similarity (e-value)*	*Identity (%)*	Gene Size **	*Blast alignment*	*Primer sequence*
GhACT4	AY305726	At5g09810	Actin gene family	6.90E-194	86%	1700	1013	TTGCAGACCGTATGAGCAAG/ATCCTCCGATCCAGACACTG
GhEF1α 5	DQ174254	At5g60390	Elongation Factor 1-alpha	5.30E-225	85%	1764	1193	TCCCCATCTCTGGTTTTGAG/CTTGGGCTCATTGATCTGGT
*GhFBX6	DR463903	At5g15710	F-box family protein	2.30E-93	79%	1884	567	TGCCTGCAGTAAATCTGTGC/GGGTGAAAGGGTTTCCAAAT
*GhPP2A1	DT545658	At1g59830	Catalytic subunit of protein phosphatase 2A	3.30E-110	77%	1301	675	GATCCTTGTGGAGGAGTGGA/GCGAAACAGTTCGACGAGAT
*GhMZA	DT571956	At5g46630	Clathrin adaptor complexes medium subunit family protein	1.40E-131	82%	1853	755	CCGTCAGACAGATTGGAGGT/AAAGCAACAGCCTCAACGAC
*GhPTB	DT574577	At3g01150	Polypyrimidine tract-binding protein homolog	1.50E-120	77%	1511	752	GGTTACCATTGAGGGTGTGG/GTGCACAAAACCAAATGCAG
*GhGAPC2	ES810306	At1g13440	Glyceraldehyde-3-phosphate dehydrogenase C-2	0.0	83%	1439	858	TCCCCATCTCTGGTTTTGAG/AACCCCATTCGTTGTCCATA
GhβTUB3	AY345606	At5g12250	Beta-tubulin	5.70E-198	80%	1696	1135	GATTCCCTTCCCTCGTCTTC/CGGTTAGAGCTCGGTACTGC
***GhUBQ14	DW505546	At4g02890	Polyubiquitin	0.0	80%	1502	510	CAACGCTCCATCTTGTCCTT/TGATCGTCTTTCCCGTAAGC
GhMADS3	ES812912	At4G18960	AGAMOUS	NA	NA	NA	NA	ATCAAGCGGATCGAAAACAC/CAACCTCAGCGTCACAAAGA
GhSEP-like1	ES827315	At1G24260	SEPALLATA3	NA	NA	NA	NA	TCCGTTCTTTGTGATGCAGA/CCATGGCTGCACTTCTGGTA

*All cotton sequences were named according the most similar ortholog locus (*GhFBX6, GhPP2A1, GhMZA, GhPTB *and *GhGAPC2 *from *Arabidopsis thaliana*) (*GhACT4, GhEF1α5 *and *GhβTUB3 *from *Gossypium hirsutum*.**Size in base pair (pb) of the coding sequence of the ortholog locus in *A. thaliana*. ***Cotton gene previously used as reference gene in qPCR [26]. NA - not applicable.

**Table 2:** Values of efficiency ± standard deviation (SD) of the primers of the housekeeping genes and average values of quantification cycle (Cq) ± standard deviation (SD) of biological replicates generated by the *Miner *to the genes of reference of *G. hirsutum*.

A	GhACT4	GhEF1α 5	GhFBX6	GhPP2A1	GhMZA	GhPTB	GhGAPC2	GhβTUB3	GhUBQ14
**Efficiency ± SD**	0.93 ± 0.026	0.97 ± 0.019	0.93 ± 0.018	0.91 ± 0.019	0.91 ± 0.021	0.93 ± 0.014	0.89 ± 0.031	0.94 ± 0.015	0.93 ± 0.022
**Plant organs**	**Cq ± SD**								
Leave	19.08 ± 0.395	19.20 ± 0.705	24.74 ± 0.191	23.66 ± 0.442	21.45 ± 1.388	23.40 ± 0.940	24.57 ± 0.663	22.29 ± 0.084	18.57 ± 0.333
Stem	17.45 ± 0.199	17.39 ± 0.150	24.99 ± 0.251	22.36 ± 0.290	21.15 ± 0.216	22.49 ± 1.592	21.65 ± 0.980	19.39 ± 0.323	16.36 ± 0.201
Branch	17.74 ± 0.648	17.25 ± 0.157	24.16 ± 0.026	22.38 ± 0.268	21.58 ± 0.092	22.20 ± 0.614	23.38 ± 0.642	19.26 ± 0.072	16.63 ± 0.187
Root	17.46 ± 0.337	18.05 ± 0.107	24.54 ± 0.991	23.06 ± 0.655	22.72 ± 0.233	22.33 ± 0.377	25.28 ± 0.236	22.45 ± 0.292	18.32 ± 0.561
Flower buds	16.70 ± 0.262	16.80 ± 0.493	23.77 ± 0.042	22.63 ± 0.141	21.71 ± 0.451	22.51 ± 1.088	24.09 ± 0.936	21.73 ± 0.174	18.20 ± 0.323
Fruits	16.25 ± 0.273	16.71 ± 0.188	24.07 ± 0.712	22.60 ± 0.181	21.46 ± 0.240	22.69 ± 0.241	24.18 ± 0.160	19.17 ± 0.135	16.51 ± 0.193
**B**	**GhACT4**	**GhEF1α 5**	**GhFBX6**	**GhPP2A1**	**GhMZA**	**GhPTB**	**GhGAPC2**	**GhβTUB3**	**GhUBQ14**
**Efficiency ± SD**	0.96 ± 0.015	0.95 ± 0.014	0.94 ± 0.015	0.92 ± 0.017	0.94 ± 0.020	0.93 ± 0.022	0.88 ± 0.024	0.94 ± 0.017	0.94 ± 0.013
**Flower buds**	**Cq ± SD**								
Floral meristem	16.84 ± 0.34	16.14 ± 0.57	23.76 ± 0.44	21.78 ± 0.73	20.94 ± 0.39	21.60 ± 0.33	24.98 ± 0.26	19.89 ± 0.32	17.31 ± 0.78
Flower bud 2 mm	20.61 ± 1.78	24.70 ± 1.59	27.93 ± 1.34	25.37 ± 1.90	25.52 ± 3.07	27.26 ± 2.27	28.49 ± 2.41	24.70 ± 1.59	21.16 ± 1.85
Flower bud 4 mm	18.53 ± 0.92	23.49 ± 0.96	25.62 ± 1.32	24.24 ± 1.11	21.94 ± 0.08	23.97 ± 1.54	27.60 ± 0.84	23.49 ± 0.96	19.04 ± 1.30
Flower bud 6 mm	15.76 ± 0.14	20.37 ± 0.24	23.41 ± 0.10	22.01 ± 0.10	20.81 ± 0.14	21.65 ± 0.21	21.03 ± 0.64	20.37 ± 0.24	16.23 ± 0.51
Flower bud 7 mm	17.17 ± 1.19	20.90 ± 0.99	24.22 ± 1.26	22.47 ± 1.10	22.55 ± 0.56	22.47 ± 0.91	21.69 ± 1.26	20.90 ± 0.99	16.99 ± 1.08
Flower bud 8 mm	16.44 ± 0.74	20.54 ± 0.18	24.34 ± 0.66	22.09 ± 0.84	21.07 ± 1.21	22.64 ± 0.78	20.98 ± 0.49	20.54 ± 0.18	16.70 ± 0.38
Flower bud 10 mm	18.06 ± 0.71	22.01 ± 1.45	26.09 ± 0.16	23.56 ± 1.54	21.68 ± 1.20	23.36 ± 0.89	22.04 ± 1.76	22.01 ± 1.45	17.38 ± 1.15
Flower bud 12 mm	15.30 ± 0.64	19.33 ± 0.83	24.03 ± 0.52	21.69 ± 0.13	20.03 ± 0.65	21.54 ± 0.62	21.41 ± 0.96	19.51 ± 0.77	15.98 ± 0.45
**C**	**GhACT4**	**GhEF1α 5**	**GhFBX6**	**GhPP2A1**	**GhMZA**	**GhPTB**	**GhGAPC2**	**GhβTUB3**	**GhUBQ14**
**Efficiency ± SD**	0.97 ± 0.021	0.92 ± 0.029	0.94 ± 0.017	0.82 ± 0.019	0.92 ± 0.024	0.91 ± 0.031	0.88 ± 0.032	0.93 ± 0.009	0.96 ± 0.024
**Floral organs**	**Cq ± SD**								
Carpels	17.34 ± 0.52	17.16 ± 1.18	24.11 ± 0.73	22.31 ± 0.66	20.85 ± 0.40	21.93 ± 0.77	22.14 ± 1.60	21.20 ± 0.28	16.12 ± 0.63
Stames	16.87 ± 0.29	16.08 ± 0.19	24.37 ± 0.09	22.12 ± 0.59	21.59 ± 0.31	21.78 ± 0.70	22.76 ± 0.53	21.33 ± 0.20	17.77 ± 0.29
Sepals	16.33 ± 0.39	15.82 ± 0.63	23.08 ± 0.36	21.96 ± 0.47	20.66 ± 0.19	21.50 ± 0.18	23.24 ± 0.12	20.31 ± 0.20	16.17 ± 0.85
Petals	18.08 ± 2.00	18.55 ± 2.52	25.39 ± 1.37	23.17 ± 0.79	22.65 ± 1.72	23.51 ± 1.56	24.09 ± 0.13	21.25 ± 1.93	18.51 ± 1.99
Pedicels	16.56 ± 0.19	16.11 ± 0.32	25.02 ± 0.85	23.69 ± 0.11	22.52 ± 0.92	23.28 ± 0.72	22.25 ± 0.56	21.60 ± 0.08	16.28 ± 0.33
**D**	**GhACT4**	**GhEF1α 5**	**GhFBX6**	**GhPP2A1**	**GhMZA**	**GhPTB**	**GhGAPC2**	**GhβTUB3**	**GhUBQ14**
**Efficiency ± SD**	0.96 ± 0.019	0.94 ± 0.17	1.01 ± 0.012	0.94 ± 0.017	1.01 ± 0.018	0.98 ± 0.014	0.96 ± 0.018	0.94 ± 0.026	0.93 ± 0.020
**Fruits**	**Cq ± SD**								
Fruits 10-15 mm	16.78 ± 0.74	18.56 ± 1.36	26.33 ± 0.30	23.43 ± 1.00	22.44 ± 0.65	24.13 ± 0.57	27.85 ± 0.51	20.67 ± 0.27	17.52 ± 0.15
Fruits 16-20 mm	17.27 ± 0.19	18.49 ± 1.17	26.64 ± 0.93	22.78 ± 1.10	20.89 ± 0.07	23.12 ± 0.48	26.79 ± 0.70	19.61 ± 0.42	17.28 ± 0.26
Fruits 21-30 mm	17.39 ± 0.47	18.89 ± 0.14	26.09 ± 0.75	23.34 ± 0.21	21.45 ± 0.28	22.75 ± 0.98	27.39 ± 0.67	20.14 ± 1.30	17.17 ± 0.18
Fruits >30 mm	19.89 ± 1.58	20.89 ± 1.78	29.17 ± 2.12	24.61 ± 0.72	23.06 ± 0.72	24.70 ± 0.46	26.94 ± 2.49	20.64 ± 1.37	18.70 ± 1.15

The values of efficiency of primers were generated for each experimental situation (A-plant organs, B-flower buds, C-floral organs and D-fruit).

Polymerase chain reactions were carried out in an optical 96-well plate with a *Chromo4 Real time PCR Detector *(BioRad) sequence detection system, using SYBR^®^Green to monitor dsDNA synthesis. Reaction mixtures contained 10 μL of diluted cDNA (1:50), 0.2 μM of each primer, 50 μM of each dNTP, 1× PCR Buffer (Invitrogen), 3 mM MgCl2, 2 μL of SYBR^®^Green I (Molecular Probes) water diluted (1:10000), and 0.25 units of Platinum Taq DNA polymerase (Invitrogen), in a total volume of 20 μL. Reaction mixtures were incubated for five minutes at 94°C, followed by 40 amplification cycles of 15 s at 94°C, 10 s at 60°C and 15 s at 72°C. PCR efficiencies and optimal quantification cycle threshold (Cq values were estimated using the online Real time PCR *Miner *tool [29]. For all reference and MADS-box genes studied, two independent biological samples of each experimental condition were evaluated in technical triplicates.

### Databases and procedures for searching Cotton MADS-box sequences

The primary data source for this work was clustered gene sequences of the Cotton Genome Database (U.S. Department of Agriculture, Agricultural Research Service CottonDB - http://www.cottondb.org.). In order to search for MADS-box sequences, a MADS-box consensus sequence was used. This consensus was generated by the COBBLER program (COnsensus Biasing By Locally Embedding Residues, http://blocks.fhcrc.org/blocks/cobbler.html) from all identified MADS-box amino acid sequences "MGRKKIEIKRIENKTNRQVTFSKRRNGLFKKAHELSVLCDAEV ALIVFSPSGrlyeyannni" [30]. Searches were conducted using the tBLASTN algorithm with the BLOSUM62 scoring matrix [31]. All sequences that exhibit a significant alignment (E-value of ≤ 7 × 10^-13^) with the consensus were retrieved from Unigene http://www.ncbi.nlm.nih.gov/UniGene/UGOrg.cgi?TAXID=3635 in the Cotton Genome Database http://cottondb.org/cdbhome.html. All retrieved sequences were then re-inspected for occurrence of MADS conserved motif using the *InterProScan *http://www.ebi.ac.uk/InterProScan/ and *PRODOM *http://prodom.prabi.fr/prodom/current/html/form.php programs. Multiple alignments with complete sequences or domains were conducted using the *CLUSTALW *program using default parameters and then manually revised [32]. Phylogenetic trees were constructed using pairwise distance matrices for neighbor-joining method [33] and p-distance on the *Mega *4.1 program [34]. Assessment of node confidence was done by means of 1,000 bootstrap replicates.

### Analysis of gene expression stability

Expression levels of the nine housekeeping genes in all the sample pools were determined by the number of cycles (Cq) needed for the amplification related fluorescence to reach a specific threshold level of detection. Cq values were converted in *qBase *software v1.3.5 [35] into non-normalized relative quantities, corrected by PCR efficiency, using the formula Q = E^ΔCq ^where E is the efficiency of the gene amplification and ΔCq is the sample with the lowest expression in the data set minus the Cq value of the sample in question. These quantities were imported into *geNorm *v3.5 [1] and *NormFinder *[12] analysis tools, which were used as described in their manuals. Data of biological replicates were analyzed separately in both programs.

## Results

In order to compare the expression levels of target genes in different tissues at the same time, it is crucial to normalize all the samples by the same set of reference genes. For the evaluation of potential reference, a well known housekeeping gene, poly-ubiquitin (*GhUBQ14*), was included in the qPCR experiments [26]. We selected eight new candidates to housekeeping genes (*GhACT4, GhEF1α5, GhFBX6, GhPP2A1, GhMZA, GhPTB, GhGAPC2, GhβTUB3*) in *G. hirsutum*. These genes are putative homologues of eight *Arabidopsis *genes included in the list of 27 best reference genes for qPCR analysis (Table 1) [6]. For the selection of the putative cotton housekeeping genes, we searched in the Cotton DB for homologues to the *Arabidopsis *referenced genes, only eight candidates that showed very high similarities (E-value > 1e-75) were included in the final list. The eight genes found in the cotton databanks belong to different functional classes based on *Arabidopsis *sequence information, which reduce the chances of co-regulated expression occurrence among these genes (Table 1). The gene name, accession number, *A. thaliana *homologue locus, *A. thaliana *annotation, similarity end identity, gene size, and primer sequence, are provided in Table 1. The nine cotton candidate reference genes were evaluated for gene expression stability by qPCR in a set of 23 cotton samples grouped into five different experimental sets. The first experimental set was composed of plant organs: leaves, stem, branch, root, flower buds (RNA pools of stages 2 to 12 mm) and fruits (RNA pools of stages 10 to 15 to fruits larger than 30 mm). The second set included floral meristem and size selected flower buds, based on their diameter of 2, 4, 6, 7, 8, 10 and 12 mm. The third experimental set was composed of the floral verticils: sepal, petal, stamen, carpel and pedicel. The fourth experimental set consists of four stages of fruit development based on it diameter: 10 to 15 (1), 16 to 20 (2), 21 to 30 (3) and larger than 30 mm (4). Finally, in the fifth set, we included all the tissues samples used in this study (23 distinct biological samples).

Total RNA was isolated from different tissue samples and reverse transcribed. The RNA quality for all samples was checked by gel eletrophoresis analisys and spectrophotometric assays (data not shown). Within a biological replicate, for a tissue sample, the same cDNA pool was used for qPCR analysis of each of the nine genes using gene-specific primers. qPCRs were performed in triplicate for each of the 23 cDNA pools along with a no template control in parallel for each gene. The melting-curve analysis performed by the PCR machine after 40 cycles of amplification and agarose gel electrophoresis showed that all the 9 primer pairs amplified a single PCR product of desired size from various cDNA (results not shown). Primer efficiencies for all primer combinations were higher than 0.90 (90%) in all experimental sets. Although, two primers pairs presented efficiencies below 90% in four samples: *GhGAPC2 *in flower buds and floral and plant organs and *GhPP2A1 *in floral organs (Table 2). The mean Cq value (average of 6 values from the two biological replicates) in a tissue sample for each gene is shown in Table 2. Cq values were in the range of 15.30 and 29.17. *GhACT4*, *GhUBQ14 *and *GhEF1α5 *are the top three most expressed genes in all sets followed by *GhMZA*, *GhβTUB3, GhPP2A1 *and *GhPTB*. *GhFBX6 *and *GhGAPC2 *genes present the lowest expression levels in all samples.

**Figure 1:**
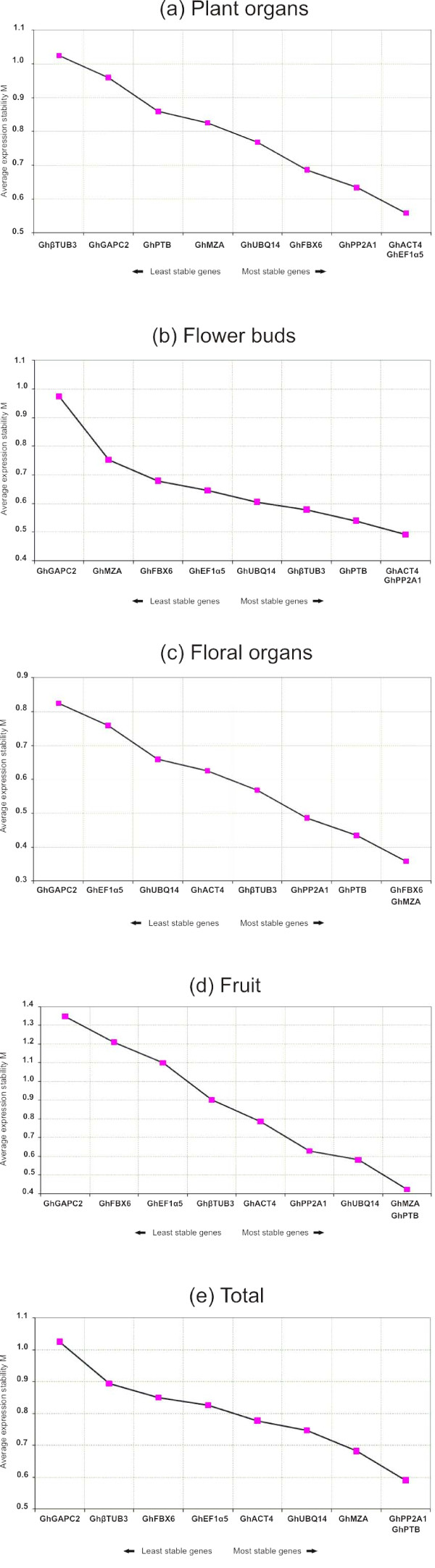
**Expression stability values (M) and ranking of the candidate reference genes as calculated by *geNORM *in al 23 cDNA samples**. Average expression stability values (M) of the reference genes were measured during stepwise exclusion of the least stable reference genes. A lower value of average expression stability, M, indicates more stable expression.

**Figure 2:**
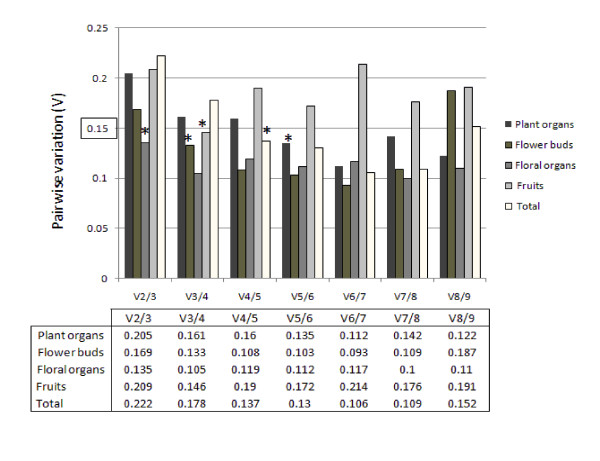
**Pairwise variation (V) to determine the optimal number of control genes for an accurate normalization**. The pairwise variation (Vn/Vn+1) was analyzed between the normalization factors NFn and NFn+1 by the *geNorm *software. Asterisk indicates the optimal number of genes for normalization.

We used *geNorm *v3.5 software, to analyze the expression stability of the tested genes in all samples, and ranked them accordingly to gene stability measure (M). The M value is obtained by the use of relative expression values for each cDNA sample as input for the *geNorm *algorithm based on the geometric averaging of multiple control genes and mean pairwise variation of a gene from all other control genes in a given set of samples. Therefore, genes with the lowest M values have the most stable expression. The results obtained with *geNorm *algorithm are presented in the Figure 1 and summarized in Table 3. The *geNorm *algorithm also determines the pairwise variation *Vn*/*n *+ 1, which measures the effect of adding further reference genes on the normalisation factor (that is calculated as the geometric mean of the expression values of the selected reference genes). It is advisable to add additional reference genes to the normalisation factor until the added gene has no significant effect. Vandesompele *et al*. (2002) used 0.15 as a cut-off value, below which the inclusion of an additional reference gene is not required. Pairwise variation analysis (Figure 2) showed that the ideal number of reference genes may be different for distinct set of samples. For instance, for the normalization of the floral organ set, only two genes are necessary. On the other hand, five genes are required for the normalization of the plant organ set. When evaluating all the pairwise variation, the least stable housekeeping gene was *GhGAPC2 *followed by *GhβTUB3 *since they significantly increased the pairwise variation during the whole assay by increasing the V value as shown in Figure 2. However, Vandesompele and collaborators recommend the use of at least three reference genes whenever this result obtained in our analysis is observed [1].

**Table 3:** Candidates genes ranked according to their expression stability estimated using *geNorm *algorithm after stepwise exclusion of the least stable reference gene

Plant organs		Flower buds		Floral organs		Fruits		Total	
**Ranking**	**Stability value (M)**	**Ranking**	**Stability value (M)**	**Ranking**	**Stability value (M)**	**Ranking**	**Stability value (M)**	**Ranking**	**Stability value (M)**

GhACT4	0.558	GhACT4	0.491	GhFBX6	0.32	GhMZA	0.422	GhPP2A1	0.59
GhEF1α5	0.558	GhPP2A1	0.491	GhMZA	0.32	GhPTB	0.422	GhPTB	0.59
GhPP2A1	0.634	GhPTB	0.539	GhPTB	0.396	GhUBQ14	0.58	GhMZA	0.682
GhFBX6	0.686	GhβTUB3	0.578	GhPP2A1	0.433	GhPP2A1	0.628	GhUBQ14	0.747
GhUBQ14	0.768	GhUBQ14	0.604	GhβTUB3	0.519	GhACT4	0.785	GhACT4	0.777
GhMZA	0.824	GhEF1α5	0.644	GhACT4	0.595	GhβTUB3	0.901	GhEF1α5	0.825
GhPTB	0.859	GhFBX6	0.678	GhUBQ14	0.682	GhEF1α5	1.09	GhFBX6	0.85
GhGAPC2	0.959	GhMZA	0.752	GhEF1α5	0.739	GhFBX6	1.21	GhβTUB3	0.894
GhβTUB3	1.024	GhGAPC2	0.973	GhGAPC2	0.821	GhGAPC2	1.34	GhGAPC2	1.024

Stability values are listed from the most stable genes to the least stable.

In addition, to the analysis by *geNorm *we also evaluated the data with *NormFinder *algorithm (Table 4). Differentially to *geNorm*, *NormFinder *takes into account intra- and intergroup variations for normalization factor (NF) calculations. When the outcome of *geNorm *and *NormFinder *are compared few, but relevant, differences are observed (Table 5). These discrepancies between the results are expected since the *geNorm *and *NormFinder *are based on distinct statistical algorithms.

**Table 4:** Cotton reference genes for normalization and their expression stability values calculated by the *NormFinder *software

Plant organs		Flower buds		Floral organs		Fruits		Total	
**Ranking**	**Stability value**	**Ranking**	**Stability value**	**Ranking**	**Stability value**	**Ranking**	**Stability value**	**Ranking**	**Stability value**
GhPP2A1	0.24	GhACT4	0.233	GhFBX6	0.179	GhMZA	0.093	GhPP2A1	0.277
GhUBQ14	0.359	GhPP2A1	0.326	GhMZA	0.266	GhPTB	0.162	GhUBQ14	0.352
GhMZA	0.375	GhUBQ14	0.339	GhPTB	0.278	GhUBQ14	0.183	GhACT4	0.362
GhEF1α5	0.379	GhPTB	0.361	GhACT4	0.3	GhPP2A1	0.189	GhMZA	0.364
GhPTB	0.564	GhEF1α5	0.367	GhPP2A1	0.302	GhACT4	0.268	GhPTB	0.37
GhFBX6	0.578	GhβTUB3	0.368	GhβTUB3	0.352	GhGAPC2	0.506	GhEF1α5	0.445
GhACT4	0.595	GhFBX6	0.463	GhUBQ14	0.479	GhβTUB3	0.561	GhFBX6	0.464
GhGAPC2	0.657	GhMZA	0.532	GhEF1α5	0.503	GhEF1α5	0.591	GhβTUB3	0.481
GhβTUB3	0.721	GhGAPC2	0.969	GhGAPC2	0.58	GhFBX6	0.647	GhGAPC2	0.714
									
Best combination	Stability value	Best combination	Stability value	Best combination	Stability value	Best combination	Stability value	Best combination	Stability value

GhUBQ14 and GhPP2A1 0.180		GhACT4 and GhUBQ14 0.222		GhACT4 and GhFBX6 0.187		GhMZA and GhPTB 0.109		GhPP2A1 and GhUBQ14 0.221	

Stability values are listed from the most stable genes to the least stable.

**Table 5:** Best combination of reference genes based on *geNorm *and *NormFinder *programs

Experimental sets				
**Plant organs**	**Flower buds**	**Floral organs**	**Fruits**	**Total**

GhUBQ14	GhACT4	GhACT4	GhMZA	GhPP2A1
GhPP2A1	GhUBQ14	GhFBX6	GhPTB	GhUBQ14
GhACT4				

Stability values are listed from the most stable genes to the least stable.

**Figure 3:**
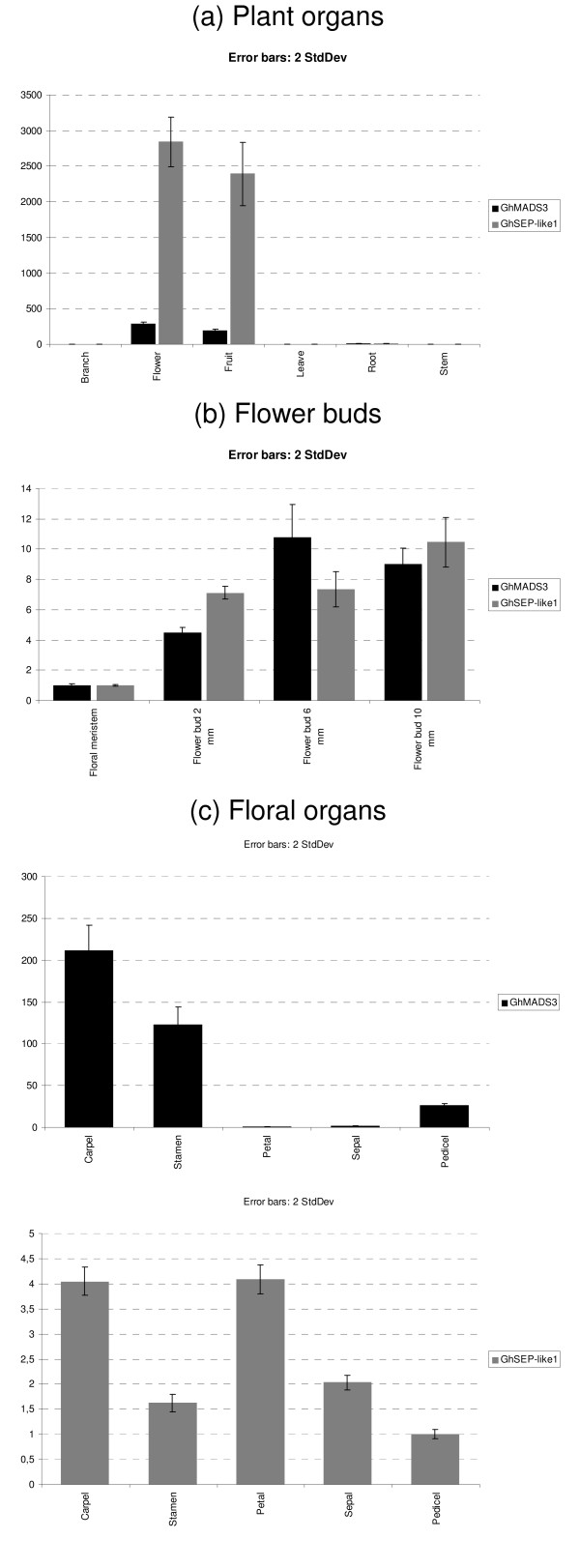
**Relative mRNA levels of *GhMADS3 *and *GhSEP-like1 *mRNA in the different plant organs (a), during the flower development (b) and in the floral organs (c)**. Cq and amplification efficiency values were processed with the *qBase *software. Normalization was performed using the best combination of reference genes recommended by *NormFinder *program to each experimental set. The combination of *GhUBQ14 *and *GhPP2A1 *were used as internal control for plant organs (a), *GhACT4 *and *GhUBQ14 *for flower buds (b) and *GhACT4 *and *GhFBX6 *for floral organs (c).

To assess the validity of the procedure for the selection of control genes detailed above, the relative expression level of two cotton genes that belong to MADS-box family were inspected. After the search in Cotton db using the MADS-box consensus sequence, 18 ESTs were found with high similarity to MIKC MADS box family (E-value ≤ 7 × 10^-13^) (Data not shown). The reduced number of cotton MIKC type genes is expected since the ESTs sequencing efforts in cotton are very limited when compared to other species such as *Arabidopsis *and rice. In spite of the low number of MADS-box genes, the phylogenetic analysis identified good candidates to homologous genes of *Arabidopsis AGAMOUS *(*AG*) and *SEPALLATA3 *(*SEP3*) (data not shown). The homologue of *AG*, was previously characterized by RT-PCR and named *GhMADS3 *[36]. RT-PCR analysis suggests that *GhMADS3 *expression is restricted to stamens and carpels. Ectopic expression in *Nicotiana tabacum *L. indicates that it is the cotton orthologous gene to *AG *[36]. The *Arabidopsis thaliana SEP3 *is expressed in the three inner whorls of organs throughout flower development, but there is no information of the putative homologue of cotton (*GhSEP-like1*), identified by our phylogenetic analysis [37]. The expression of *GhMADS3 *and *GhSEP-like1 *was estimated in different plant organs, during flower development and in the floral organs of 6 mm flower buds. The qPCR analysis employed the control genes recommended by *NormFinder *program for the normalization of gene expression. The analysis revealed that *G. hirsutum GhMADS3 *and *GhSEP-like1 *genes very similar expression profiles of *AG *and *SEP3 *genes from *Arabidopsis *(Figure 3). However, we also observed unexpected expression patterns: *GhSEP-like1 *is expressed in cotton fruits and the *GhMADS3 *in pedicels of 6 mm flower buds.

## Discussion

The qPCR is broadly accepted as the method of choice for accurate and sensitive quantification of gene transcript levels, even for those genes whose transcript levels are low. For valid qPCR analysis, accurate normalization of gene expression against an appropriate internal control is required. The ideal control gene should have similar expression regardless of experimental conditions, including different cell types, developmental stages, and/or sample treatment. However, no one gene has a stable expression under every experimental condition, as numerous studies reported that expression of housekeeping genes can also vary considerably with experimental conditions. Consequently, normalization of gene expression with a single reference gene can trigger erroneous data and, consequently, misinterpretation of experiment results. Therefore, it is necessary to validate the expression stability of a control gene under specific experimental conditions prior to its use in qPCR normalization.

Normalisation with multiple reference genes is becoming the golden standard, but reports that identify such genes in plant research are limited [3,4,17,18,38,39], even though algorithms are available to test the expression stability of candidates [1,12,13] and a number of candidate reference genes for *Arabidopsis *have been proposed [6]. To obtain a solid basis for normalization of our gene expression data when studying the flower development in cotton, we evaluated the expression stability of nine candidate reference genes, including one traditional "housekeeping" gene in five different experimental sets. Candidate genes were selected according to the level of DNA sequence similarity to genes previously identified as reference genes in *Arabidopsis *and cotton. This strategy has been successful in finding good reference genes in other species such as grape [39] and it was already employed by our group in coffee and *B. brizantha *[17,18]. Another strategy used to identify bona fide qPCR reference genes is to check housekeeping genes previously used in Northern and RT-PCR studies [40,41]. However, it has be shown that the expression of traditional reference genes may vary enormously depending on the test condition [6]. In cotton, Tu and collaborators tested six putative constitutive genes (*Histone3, UBQ7, Actin, Cyclophilin, Gbpolyubiquitin-1 *and *Gbpolyubiquitin-2*), two of them (*Gbpolyubiquitin-1 *and *Gbpolyubiquitin-2*) from previously published data [42]. In contrast to the present work, roots, floral stages and verticils samples were not included in the final set of samples [41]. The reference genes evaluation was performed using exclusively *geNorm *and the value obtained for the pairwise variation with the best control genes was above the cut-off value of 0.15 suggested by Vandesompele *et al*. [1]. Moreover, the expression in the fiber developmental series of the all six putative reference genes varied greatly, hampering their use for qPCR [41].

We elected the *NormFinder *as the preferential method for the selection of the best references genes since it considers intra- and inter-group variations for the normalization factor (NF). However, *geNorm *was also important to compose the final set of references genes for the experimental conditions tested in this work. Our analysis has shown that each experimental condition tested demands a specific set of reference genes (Table 3 and 4). This result emphasizes the importance of reference genes validation for each experimental condition, especially when samples belong to very different groups, e.g. different organs.

When plant organs and all samples were tested, *GhUBQ14 *and *GhPP2a1 *were considered the most appropriate reference genes. *GhUBQ14 *and *GhPP2a1 *should avoid error transferences since *NormFinder *chose them as the best combination of genes. *NormFinder *chose *GhACT4 *and *GhUBQ14 *as the best combination of two genes in flower buds. Both programs ranked *GhACT4 *as the most stable gene, conferring higher robustness to the NF. Our analyses of different floral organs revealed that *GhACT4 *and *GhFBX6 *are the most appropriated genes for qPCR normalization, since they represent the best combination of genes considered by *NormFinder *to improve NF. *GhFBX6 *was ranked by both algorithms as the most stable gene in the floral organs set. Finally, fruit development *GhMZA *was considered as the most stable gene in both the *NormFinder *and *geNorm *programs, and *NormFinder *chose *GhMZA *and *GhPTB *as the best combination of genes.

The *GhACT4, GhEF1α5, GhFBX6, GhPP2A1, GhMZA, GhPTB, GhGAPC2, GhβTUB *genes were identified as novel reference genes in *A. thaliana *through microarray experiments and were validated by qPCR [7]. Among them, *GhGAPC*2 gave poor results in our analysis in cotton. *GhUBQ14*, a traditional reference gene in cotton [26] was well evaluated by *NormFinder *ranking in the best combination in three of the five experimental sets. Although, evaluations of a traditional reference genes by the same procedures used in this work not always give support to their frequent use. For instance, *UBQ10 *gene shows highly stable expression in *Arabidopsis *[6] whereas its putative homologue has been shown unsuitable for normalization of different tissues at different developmental stages in rice and soybean [4,43].

Other commonly used housekeeping gene, *GhβTUB*, displayed inappropriate expression variability limiting its use as internal control in cotton. A similar result was also observed for the *β-tubulin *of *B. brizantha *when male and female reproductive tissues, spikelets, roots and leaves were evaluated [17]. On the other hand, *β-TUB *is one of most stably expressed genes in poplar (*Populus ssp*) tissue samples among the 10 reference genes tested [10]. *GAPDH*, another traditional reference gene, was considered the most appropriate reference gene when coffee leaves drought-stressed vs. control plants and different coffee cultivar leaves were analyzed [18]. Taken together, these results suggest that the housekeeping genes are regulated differently in different plant species and may exhibit differential expression patterns. This may partly be explained by the fact that housekeeping genes are not only implicated in the basal cell metabolism but also may participate in other cellular functions [11].

The programs employed to evaluate reference genes in our study (*geNorm *and *NormFinder*) use the same input data, i.e. non-normalized relative quantities, and Cqs need to be transformed considering primer pair efficiencies. In our experience, it is crucial to evaluate primer pair efficiencies for each sample tested since primer efficiency varies depend on the according to biological sample. The importance of this step can be well illustrated by the primer efficiency variation of *GhGACP2 *in flower buds compared to fruits (Table 2).

The values of Cq presented here should not be considered alone, but they may help in the selection of best combination of reference genes when there is previous data about target gene expression levels. Similar expression levels of the reference and target genes are considered an important issue regarding qPCR normalization [1]. Indeed, references genes with excessively high/low expression levels compared to target genes can trigger problems for data analysis [44,45].

As suggested by Remans and collaborators [7], biological replicates were submitted to *geNorm *and *NormFinder *as independent samples. This procedure increased the credibility of the most suitable cotton reference genes because it takes into account possible variations in reference gene expression that are not due to different treatments, but intrinsic to the gene itself.

To illustrate the suitability of the reference genes revealed in the present study, two putative cotton homologues to *AG *and *SEP3 (GhMADS3 and GhSEP-like1) *had their expression profile evaluated in different plant organs, during flower development and in floral organs at flower buds of 6 mm (Figure 3). As it is observed to *AG *and *SEP3*, the *GhMADS3 *and *GhSEP-like1 *genes are highly expressed in flower buds, but *GhSEP-like1 *also shows a high expression in fruits. *GhMADS3 *also is expressed in higher levels after stage of 2 mm and throughout cotton flower development. The low expression of *GhMADS3 *in floral meristem is expected as well a high expression level in stamen and carpels of 6 mm flower bud. The *AG *gene is expressed in few cells during the initial flower development to establish organ identity and is also important at later stages of stamens and carpels development [46,47]. The *GhMADS3 *expression observed in pedicels may be the result of contamination of material derived from carpels. These two organs are merged, which hamper a perfect separation during flower bud dissection to collect the samples. Our analysis of *GhMADS3 *expression by RT-qPCR refined the information of the previous study adding accuracy, spatial and temporal information to the expression during floral development [36]. In addition, it revealed that this *GhMADS3 *is also expressed in cotton fruits (Figure 3).

The high expression in fruits of *GhSEP-like1 *contrast to the spatial and developmental expression pattern of *SEP3 *in Arabidopsis, former *AGL9 *(Figure 3) [37]. *SEP3 *is expressed in all *Arabidopsis *flower verticils throughout development but no transcripts are found in siliques. However, *PPERSEP3*, a putative *Prunus persica *homologue of *SEP3*, is expressed during fruit development [48]. In addition, *GhMADS4 *and *GhMADS7*, genes from *AGAMOUS *subclass in cotton, are also expressed during fiber development [23]. Taking together, these results suggest that besides flower identity *SEP3 *and *AG-like *genes in cotton may be involved in an additional developmental process during fruit development.

## Conclusion

This work constitutes the first in-depth study to validate the optimal control genes for the quantification of transcript levels in different cotton organs and during flower and fruit development. The use of the new cotton reference genes combined with size collected flower buds and floral organ dissection allowed a precise spatial and temporal characterization of two MADS-box genes in cotton plants. In summary, the new cotton reference genes will enable more accurate and reliable normalization of qPCR results for gene expression studies in this important crop plant.

## Authors' contributions

SA and SMN were responsible for the experiments, RNA sample preparation, RT-qPCR data analyses and drafting the manuscript. OS and MF G-S contributed with sample preparation and study design. MA-F participated as supervisor in the study design, analyses and writing. All authors read and approved the final manuscript.

## Supplementary Material

Additional file 1 **List of samples of *G. hirsutum *flower and fruit used in this study with the respective major biological events observed**. We prepared paraffin transverse sections of cotton flower buds in order to visualize the changes that occurred at the cellular level. Click here for file

Additional file 2 **Values of efficiency ± standard deviation (SD) of the primers and average values of quantification cycle (Cq) ± standard deviation (SD) of biological replicates generated by the *Miner *to the MADS-box genes of *G. hirsutum***. The values of efficiency of primers were generated for each experimental situation (A-plant organs, B-flower buds and C-floral organs). Click here for file
