# Supplementary material for: Identification and evaluation of new reference genes in Gossypium hirsutum for accurate normalization of real-time quantitative RT-PCR data
Source: BMC Plant Biol. 2010 Mar 21;10:49. doi: 10.1186/1471-2229-10-49 (PMC2923523; doi:10.1186/1471-2229-10-49)
Supplement: Additional file 1 — List of samples of G. hirsutum flower and fruit used in this study with the respective major biological events observed. We prepared paraffin transverse sections of cotton flower buds in order to visualize the changes that occurred at the cellular level. [file 1471-2229-10-49-S1.PDF]

Sample	Flower/Fruit diameter	Major Events
	**Flower**	
1	2 mm	Formation of pollen mothers cells in the anthers
2	4 mm	PMCs in prophase and anther tapetum cell layer is deferentiated
3	6 mm	Round unicellular microspores are found in the locules. Microspore sculpuctured
4	7 mm	exine is formed
5	8 mm	Tapetum degeneration initiated
6	10 mm	
7	12 mm	Pollen mitotic divisions occur and tapetum degenerates
	**Fruit**	
1	10 to 15 mm	Undeveloped cotyledons
2	15 to 20 mm	Cotyledons developing
3	20 to 30 mm	Cotyledons developed and black layer forming
4	Larger than 30 mm	Mature black layer
