# Supplementary material for: Identification and evaluation of new reference genes in Gossypium hirsutum for accurate normalization of real-time quantitative RT-PCR data
Source: BMC Plant Biol. 2010 Mar 21;10:49. doi: 10.1186/1471-2229-10-49 (PMC2923523; doi:10.1186/1471-2229-10-49)
Supplement: Additional file 2 — Values of efficiency ± standard deviation (SD) of the primers and average values of quantification cycle (Cq) ± standard deviation (SD) of biological replicates generated by the Miner to the MADS-box genes of G. hirsutum. The values of efficiency of primers were generated for each experimental situation (A-plant organs, B-flower buds and C-floral organs). [file 1471-2229-10-49-S2.PDF]

A	GhMADS3	GhSEP-like1	GhPP2A1	GhUBQ14
**Efficiency ± SD**	0.98 ± 0.018	0.97 ± 0.015	0.98 + 0.017	0.96 + 0.017
**Plant organs**		**Ct ± SD**		
Leave	26.47 ± 0.328	29.85 ± 0.503	24.37 ± 0.074	18.10 ± 0.179
Stem	27.78 ± 0.043	30.81 ± 0.248	23.78 ± 0.173	18.13 ± 0.081
Branch	27.34 ± 0.290	31.09 ± 0.522	23.58 ± 0.215	17.22 ± 0.229
Root	24.72 ± 0.419	28.46 ± 0.165	24.29 ± 0.345	18.83 ± 0.252
Flower bud	19.09 ± 0.049	19.35 ± 0.131	23.33 ± 0.074	17.64 ± 0.091
Fruit	20.79 ± 0.366	20.73 ± 0.537	24.47 ± 0.325	18.57 ± 0.328
**B**	**GhMADS3**	**GhSEP-like1**	**GhACT4**	**GhUBQ14**
**Efficiency ± SD**	0.95 ± 0.018	1.01 ± 0.019	0.96 + 0.018	0.95 + 0.021
**Flower buds**		**Ct ± SD**		
Floral meristem	22.94 ± 0.096	22.71 ± 0.558	17.02 ± 0.212	17.32 ± 0.781
Flower buds 2mm	19.26 ± 0.391	19.38 ± 0.063	15.94 ± 0.247	17.14 ± 0.093
Flower buds 6mm	19.32 ± 0.270	20.10 ± 0.126	16.69 ± 0.202	18.12 ± 0.400
Flower buds 10mm	18.47 ± 0.112	19.14 ± 0.425	16.22 ± 0.565	17.47 ± 0.315
**C**	**GhMADS3**	**GhSEP-like1**	**GhACT4**	**GhFBX6**
**Efficiency ± SD***	0.96 ± 0.027	0.95 ± 0.030	1.00 + 0.016	0.90 + 0.020
**Floral organs**		**Ct ± SD**		
Carpels	16.57 ± 0.252	18.10 ± 0.080	16.17 ± 0.184	23.24 ± 0.186
Stames	17.74 ± 0.138	20.12 ± 0.157	15.99 ± 0.113	24.67 ± 0.193
Sepals	23.93 ± 0.127	19.50 ± 0.194	16.49 ± 0.131	23.33 ± 0.127
Petals	24.46 ± 0.701	18.84 ± 0.182	17.49 ± 0.20	23.49 ± 0.167
Pedicels	20.85 ± 0.283	21.67 ± 0.178	17.33 ± 0.396	24.61 ± 0.371

*The values of efficiencies of the primers were generated for each experimental situation (A, B and C).
